# Sensing and Responding of Cardiomyocytes to Changes of Tissue Stiffness in the Diseased Heart

**DOI:** 10.3389/fcell.2021.642840

**Published:** 2021-02-26

**Authors:** Juliane Münch, Salim Abdelilah-Seyfried

**Affiliations:** ^1^Institute of Biochemistry and Biology, University of Potsdam, Potsdam, Germany; ^2^Institute of Molecular Biology, Hannover Medical School, Hannover, Germany

**Keywords:** mechanobiology, tissue stiffness, cardiomyocyte, heart regeneration, titin, collagen, agrin, extracellular matrix

## Abstract

Cardiomyocytes are permanently exposed to mechanical stimulation due to cardiac contractility. Passive myocardial stiffness is a crucial factor, which defines the physiological ventricular compliance and volume of diastolic filling with blood. Heart diseases often present with increased myocardial stiffness, for instance when fibrotic changes modify the composition of the cardiac extracellular matrix (ECM). Consequently, the ventricle loses its compliance, and the diastolic blood volume is reduced. Recent advances in the field of cardiac mechanobiology revealed that disease-related environmental stiffness changes cause severe alterations in cardiomyocyte cellular behavior and function. Here, we review the molecular mechanotransduction pathways that enable cardiomyocytes to sense stiffness changes and translate those into an altered gene expression. We will also summarize current knowledge about when myocardial stiffness increases in the diseased heart. Sophisticated *in vitro* studies revealed functional changes, when cardiomyocytes faced a stiffer matrix. Finally, we will highlight recent studies that described modulations of cardiac stiffness and thus myocardial performance *in vivo*. Mechanobiology research is just at the cusp of systematic investigations related to mechanical changes in the diseased heart but what is known already makes way for new therapeutic approaches in regenerative biology.

## Introduction

The heart is both an electrical and mechanical organ. A heartbeat is initiated when pacemaker cells at the sinus venosus produce action potentials. This triggers the contraction of cardiomyocytes, which generates the mechanical force required to move blood throughout the body. During each contractile cycle of the heart, cardiomyocytes experience various types of forces, including the hemodynamic pressure by blood, contraction-induced elongation/shortening, and passive elasticity/stiffness from the surrounding extracellular matrix (ECM). Sensitivity of cardiomyocytes to changes in these physical parameters is critical and the mechanical work of the heart has to be adjusted to the levels needed for the physiological blood-based transport of nutrients, gases, and metabolic waste products. Myocardial infarction and cardiomyopathies are characterized by cardiac remodeling, a process that includes increases of cardiomyocyte cell size (hypertrophy), fibroblast proliferation, and the deposition of ECM proteins. These changes alter tissue stiffness and extracellular mechanical stimulation of cardiomyocytes, which affects their biomechanical signaling and heart function. Although cardiomyocytes possess the machinery to sense those mechanical alterations in the diseased heart, ultimately, this does not prevent a reduced cardiac function. However, we are just beginning to understand how biomechanical signaling changes in the diseased heart affect cardiomyocytes. This knowledge promises the development of regenerative cardiac therapies. Many of the proteins covered in this review have been linked to various types of cardiomyopathies. This clearly demonstrates the relevance of cardiac mechanobiology for both normal physiology and for understanding the etiology of cardiomyopathies. Due to the brevity of this review article, we refer to other review articles that cover the disease aspects of these proteins (Zemljic-Harpf et al., 2009; Kamdar and Garry, 2016; Singh and Robbins, 2018).

Here, we review the current state and rapid advances in the field of cardiomyocyte mechanobiology. These have also contributed to a better understanding of myocardial diseases and led to the discovery that cardiac regenerative processes can be modulated by changing mechanical properties of the heart. First, we focus on cytoskeletal and extracellular factors that determine physiological myocardial stiffness. Understanding how changes of tissue stiffness affect the diseased heart requires a clear understanding of which cellular components sense alterations of extracellular mechanical stimuli and transduce these into biochemical signaling and gene expression. For instance, mechanosensitive YAP signaling is highly responsive to matrix stiffness changes in cardiomyocytes and potently regulates cardiomyocyte proliferation (Mosqueira et al., 2014; Bassat et al., 2017; Wang et al., 2020). We will report about the effects of stiffness changes on cardiomyocyte performance, such as sarcomere assembly and contraction, and discuss biomechanical cellular signaling that causes abnormal cardiomyocyte behaviors. Then, we integrate this information and discuss what approaches have been taken to modulate the ECM to improve cardiac healing. Although cardiac mechanobiology research is emerging rapidly, we only have a limited knowledge about how to modulate the ECM in the diseased heart. Our review discusses molecular mechanisms that determine and sense stiffness changes in the heart and highlights disease-related alterations.

## Intra- and Extracellular Components Determine Myocardial Stiffness

Cardiac conduction imposes a constant mechanical deformation on the myocardium. Intra- and extracellular factors contribute to tissue stiffness, a cardiac mechanical property that allows cardiomyocytes to withstand constant mechanical impact. Stiffness is defined as the resistance of an object to elongation or shortening. The measure of stiffness is the elastic modulus or Young’s modulus *E*, which describes the relation between tensile stress and axial strain of a material (unit in Pa). During the cardiac contractile cycle, the passive stiffness of the myocardium determines its maximal diastolic capacity/volume.

Cardiac stiffness changes dramatically during early life. In a comparative study, the murine heart was found to stiffen progressively between embryonic development day E2 with values of < 1 kPa until E14 with a 10-fold increase in stiffness, which is a value that was measured also in neonate and adult myocardium (Majkut et al., 2013). Concomitantly, there are comparable increases of cardiac actomyosin contractile proteins, adhesion proteins, and the calcium pump sarcoendoplasmic reticulum calcium transport ATPase (SERCA), which are all involved in the excitation and contraction coupling system of the heart (Majkut et al., 2013).

Other studies revealed that Young’s modulus values vary in different vertebrate hearts (Ward and Iskratsch, 2020). Berry et al. reported an elastic modulus of adult rat hearts of around 18 ± 2 kPa (Berry et al., 2006). Similarly, Bhana et al. revealed tissue stiffness values of 4–11.4 kPa for native neonatal rat myocardium and 11.9–46.2 kPa for adult rat myocardium. Cardiomyocytes derived from human-induced pluripotent stem cells exhibited a stiffness with a Young’s modulus of about 1.25 kPA, which decreased upon cytochalasin D-induced filamentous actin depolymerization (Pires et al., 2019).

The maintenance of physiological levels of cardiac stiffness not only determines overall ventricular diastolic function but also ensures proper cardiomyocyte functionality. When neonatal rat myocardium was cultured on substrates with a stiffness value of an adult heart, cardiomyocytes acquired cell morphologies and functions that were characteristic of a differentiated myocardium (Bhana et al., 2010). Similarly, Engler et al. found embryonic cardiomyocytes had an optimal contractility on matrices with stiffness values similar to the developing myocardium (Engler et al., 2008) and neonatal rat cardiomyocytes matured best on substrates with stiffness values of native, uninjured myocardium (Jacot et al., 2008). Hence, physiological stiffness of cardiac tissues is a crucial environmental mechanical cue that affects the development and mechanical properties of myocardial cells.

Extra- and intracellular components contribute to myocardial stiffness. On its extracellular side, cardiomyocytes are embedded within an ECM with a characteristic composition of glycosaminoglycans, proteoglycans, collagens, laminins, and fibronectins. These ECM proteins determine cell adhesion, cell motility, and contribute to outside-in signaling into cardiomyocytes (Chin et al., 2019). Strikingly, protein composition and levels of cross-linking change during development or disease, which affects the physiological stiffness of the heart (Ward and Iskratsch, 2020). Collagens are particularly important players in this context due to their ability to form thick, stiff and long fibrils, that can be densely packed to contribute to tissue stiffness in many organs (Tang, 2020). Changes in cardiac stiffness that occur during transition from neonatal to adult hearts have been attributed to the total amount of collagen and to the ratio of collagen type I versus collagen type III. Collagen type I provides rigidity while collagen type III increases elasticity (Marijianowski et al., 1994). Also, high levels of collagen crosslinking by the enzyme lysyl oxidase and high ratios of glycosylated lysine or hydroxylysine residues contribute to a reduced myocardial compliance (Ward and Iskratsch, 2020). Interestingly, lysyl oxidase upregulation and increased crosslinking enhanced cardiac stiffness and resulted in diastolic dysfunction (López et al., 2010).

Intracellular properties of cardiomyocytes also contribute to cardiac stiffness. The main regulator of intracellular stiffness of cardiomyocytes is titin, a giant protein that spans half of the sarcomere (Gregorio et al., 1999) and that underlies the myofibrillar passive tension response to stretch in striated muscle cells (Cazorla et al., 2000). Genetic alterations of the titin gene or developmental/disease-triggered posttranslational modifications affect passive myocardial stiffness. This has been extensively reviewed elsewhere (Granzier and Labeit, 2004; Krüger et al., 2009; LeWinter and Granzier, 2014; Tharp et al., 2019).

Titin comes in two isoforms within the mammalian heart (Freiburg et al., 2000). The more compliant isoform N2BA (>3.2 MDa) is predominant in the developing heart of several mammalian species and chick while the smaller and stiffer isoform N2B (3.0 MDa) is present shortly after birth. It has been suggested that titin isoform switching, together with isoform switching of troponin I and myosin, determines passive stiffness changes and functional transitions of the heart after birth (Lahmers et al., 2004; Opitz et al., 2004; Warren et al., 2004; Opitz and Linke, 2005). Direct triggers of these changes are genetic, species-specific (Krüger et al., 2006) or altered humoral factors (reviewed in Krüger and Linke, 2009).

While the overall amount of titin in cardiomyocytes is similar, the ratio of both isoforms varies in the myocardium of different mammalian species (Cazorla et al., 2000). Wu et al. examined the titin subtypes within the myocardium of the mouse ventricular wall, as well as the bovine left atrium and left ventricle, and found different ratios of N2BA and N2B. This study demonstrated that, under physiological conditions, a higher ratio of the shorter N2B isoform together with increases of collagen levels resulted in higher myocardial stiffness (Wu et al., 2000).

Microtubule networks and intermediate filaments also contribute to myocardial elasticity. Microtubules are supramolecular assemblies of α/β-tubulin heterodimers that are organized as highly dynamic filaments with multiple roles in mitosis, cell motility, or intracellular molecular transport. Within cardiomyocytes, microtubules generate resistance to the compressive load of the heart, which does not only depend on microtubule density but also on specific de-tyrosinations, which allow crosslinking with intermediate filaments. This affects myocardial stiffness and viscoelasticity (Robison et al., 2016; Ward and Iskratsch, 2020). In cardiac muscle, desmin is the predominant intermediate filament, which contributes to passive stiffness of cardiomyocytes and provides a link from the Z-disk to costameres (Ward and Iskratsch, 2020). Its filaments form a network surrounding myofibrils at the Z-band and in the intermyofibrillar space (Tokuyasu, 1983). During cardiac diseases, desmin is modified by phosphorylation or in expression levels (Ward and Iskratsch, 2020). The cytoskeletal components actin and myosin are required in cardiomyocytes to generate contractile force but play only a minor role in contributing to myocardial passive stiffness. Hence, several extra- and intracellular components, including titin and collagens, enable cardiomyocytes to maintain the physiological stiffness, which is required to sustain the mechanical pressure upon heart beating.

## Pathological Changes in Cardiac Stiffness Impair the Function of the Diseased Heart

The etiology of many cardiac pathologies has remained unresolved. However, increasing evidence points at a contribution of defective biomechanics in these diseases. These findings raise the question whether changes in mechanical properties of cardiomyocytes are causative to cardiac pathologies or whether pathologies affect biomechanical properties of cardiomyocytes. While it has been difficult to resolve the causality of events in many cardiac pathologies, increasing evidence suggests that physiological levels of cardiac stiffness are disturbed in many instances of cardiac diseases. Both, the cardiac ECM and intracellular components (titin) contribute to physiological levels of cardiac stiffness, which is disturbed in many cardiac diseases (Tharp et al., 2019). The roles of biomechanics-related cardiomyocyte gene mutations in the etiologies of cardiomyopathies have extensively been reviewed elsewhere (Brodehl et al., 2018; Ware and Cook, 2018; Tharp et al., 2019). Here, we mainly focus on cardiac diseases due to myocardial stiffness changes resulting from ischemic heart diseases, hypertension, or cardiac hypertrophy. Myocardial infarction caused by an obstruction of coronary vessels results in the massive death of cardiomyocytes. This triggers an immense inflammatory response. The adult mammalian heart lacks the capacity of myocardial regeneration. Instead, the loss of myocardium in the infarcted ischemic region causes a permanent replacement by a rigid fibrotic scar. This is crucial to preserve the structure of the heart and prevent ventricular rupture (Frangogiannis, 2019). However, fibrotic tissue also accumulates in other conditions of the heart such as pressure overload, metabolic dysfunction, and aging (Hinderer and Schenke-Layland, 2019). Cardiac fibrosis is characterized by increased expression and crosslinking of ECM proteins, which dramatically change myocardial stiffness. The main contributors to fibrotic tissue are cardiac fibroblasts, which secrete ECM proteins including collagens, fibronectin, laminin, elastin, fibrillin, proteoglycans, and glycoproteins. But fibroblasts also release enzymes such as matrix metalloproteases (MMPs) and tissue inhibitors of metalloproteinases (TIMPs), which modulate the ECM (Fan et al., 2012). Similarly, macrophages are gaining increased attention as an ECM-producing and -modulating cell type. Macrophages not only possess a crucial role in secreting MMPs but also secrete ECM proteins (O’Rourke et al., 2019; Simões et al., 2020). Different reports showed that fibrosis increases cardiac tissue stiffness. Coronary artery ligation-induced myocardial infarction in rat hearts resulted in fibrotic tissue with a Young’s modulus of 55 ± 15 kPA (Berry et al., 2006). Infarcted myocardium in sheep hearts showed increased stiffness within 1–2 weeks upon myocardial infarction but later returned to physiological levels (Gupta et al., 1994). Yamamoto et al. (2002) examined hearts from rats fed on a high-salt diet that had died from heart failure, which included hypertension, left ventricular hypertrophy, and fibrosis. Increased cardiac stiffness in those hearts was attributed to collagen accumulation and increased collagen-crosslinking rather than to hypertrophic cardiomyocytes (Yamamoto et al., 2002).

The giant molecule titin is the main intracellular regulator of passive tension in cardiomyocytes. Beside the ratio of N2B/N2BA isoforms, passive resistance of cardiomyocytes also depends on posttranslational titin modifications. Most studies reported important phosphosites within the “spring-like” I-band domain (Hidalgo et al., 2009; Hamdani et al., 2017; Tharp et al., 2019.). Increasing phosphorylations by PKCα at the proline-glutamate-valine-lysine (PEVK) spring element within the I-band domain cause increased myocardial stiffness (Hidalgo et al., 2009). In contrast, phosphorylation at the elastic I-band domain N2B-unique sequence (N2Bus) reduces titin tension and passive stiffness of cardiomyocytes (Hidalgo et al., 2009; Krüger et al., 2009, Kötter et al., 2013; Hamdani et al., 2017). In a rat model of concentric hypertrophy following pressure-overload, cardiac stiffness increased due to alterations in both aberrant extracellular collagen and reduced titin phosphorylation at the N2Bus domain. This resulted in diastolic dysfunction (Røe et al., 2017). Kötter et al. also revealed a crucial function for titin in the infarcted heart. Passive tension of cardiomyocytes, a contributor to myocardial stiffness, was increased as early as 3 days post myocardial infarction. Although the ratio of N2B/N2BA titin isoforms was unchanged at 3 days post myocardial infarction, phosphorylation on the PEVK spring element increased and phosphorylation of the N2Bus region decreased (Kötter et al., 2016). Interestingly, these titin modifications in the infarcted hearts were mediated by the inflammatory cytokine interleukin-6 (IL-6) (Kötter et al., 2016), which is expressed in the early inflammatory phase 3–72 h post myocardial infarction (Frangogiannis, 2006). Hence, the immune system contributes to the adaptation of myocardial stiffness in the infarcted heart.

The intracellular stiffness factors desmin and α-actinin are additional players in cardiac diseases. In a mouse model of diastolic dysfunction, levels of desmin and α-actinin increased at Z-disks. The authors suggested this to be a response to the increased strain to cardiomyocytes in this pathologic condition (Sheng et al., 2016), which points at a potential involvement of desmin in the cardiomyocyte response to mechanical changes. This would be a role in addition to its main function in sarcomere integrity and cell survival in several cardiac diseases (Hein et al., 2000). Indeed, the loss of desmin resulted in increased passive tissue stiffness in soleus muscle (Anderson et al., 2001). Further studies are required to understand the different roles of desmin in modulating myocardial passive stiffness versus adjusting myofibril and sarcomere integrity. In addition to the study of Sheng et al., also Sumita Yoshikawa et al. implicated actomyosin in pathological stiffness changes. Residual actin-myosin cross-bridge formation lead to increased passive cardiomyocyte stiffness in hypertrophic hearts (Sumita Yoshikawa et al., 2013). This is interesting because actomyosin networks play a rather minor role in influencing passive myocardial stiffness under physiological conditions.

The importance of physiological cardiac stiffness was also reported in zebrafish mutants that lacked caveolin-1, the main structural protein of caveolae. Caveolae are small membrane invaginations and are protective to mechanical stress by providing a membrane buffer when cells become stretched. The loss of caveolin-1 diminished caveolae formation in mice and also in the zebrafish heart (Grivas et al., 2020). Hearts that lacked caveolae showed an increased stiffness compared to control hearts and were functionally impaired (Grivas et al., 2020). This study further revealed a transient reduction in injury-induced cardiomyocyte proliferation in *caveolin-1* mutant hearts. However, overall regeneration was not affected. This was in contrast with previous observations, based on a different *caveolin-1* mutant, in which cardiomyocyte proliferation and cardiac regeneration were disturbed. Yet, mechanical properties and cardiac function were not examined in those animals (Cao et al., 2016). Findings of both studies raised the intriguing question, how modifications of physiological stiffness and/or reduced cardiac function *per se* influence regenerative capacities in zebrafish and mammalian hearts.

Changes in cardiac stiffness are hallmark characteristics of various cardiac diseases. Hearts of patients suffering from cardiac hypertrophy by pressure overload showed increased stiffness at the level of cardiomyocytes and ECM. This study demonstrated that both intra- and extracellular changes of biomechanical properties of the heart were affected in this condition (Chaturvedi et al., 2010).

Similarly, hearts of patients with hypertension and heart failure with a preserved ejection fraction (HFpEF), one of the main causes of heart failure, presented with increased myocardial passive stiffness together with ventricular remodeling and abnormalities in left ventricular diastolic function (Zile et al., 2004, 2011, 2015). This correlated with a decrease in titin phosphorylation on its PEVK spring and N2B elements and augmented total collagen deposition.

To date, we are lacking treatment options to avoid the detrimental changes in cardiac stiffness However, studies in mouse demonstrated that cardiac stiffness can be limited by exercise. In a mouse model with HFpEF, the authors examined the effects of free wheel running exercise on diastolic stiffness. This revealed a beneficial effect that was caused by beneficial phosphorylation changes in the PEVK and N2B spring elements of titin, which control stiffness properties of this important mechanical sarcomeric protein. In comparison, exercise did not decrease stiffness of the ECM. In pharmacological experiments, the authors used the drug ivabradine to lower the heart rate, which mimics one of the physiological effects of effective exercise. However, this treatment did not change the passive stiffness of the heart in the HFpEF mouse model. Taken together, this study demonstrated a beneficial effect of exercise-induced regulatory phosphorylations in titin that softens cardiac stiffness (Slater et al., 2017).

In conclusion, several reports showed that cardiac stiffness increases and cardiac functionality is severely impaired within the diseased heart (Gupta et al., 1994; Chaturvedi et al., 2010; Kötter et al., 2016; Sheng et al., 2016; Røe et al., 2017). These findings point at a vicious cycle in which increased passive stiffness of the heart impairs beneficial exercise, which again worsens cardiac stiffness due to pathological changes in ECM composition (mainly collagen) or negative regulatory modifications of titin.

Unlike most cardiac diseases characterized by increased tissue stiffness, patients suffering from dilated cardiomyopathy (DCM) present with reduced myocardial passive tension causing ventricular enlargement and impaired systolic function. DCM frequently results from genetic defects in the titin gene (reviewed in Tharp et al., 2019). However, lowered myocardial stiffness of end-stage heart failure patients suffering from non-ischemic DCM has also been attributed to an increased N2B-to-N2BA ratio. Hence, the more compliant large isoform of titin (N2BA) was augmented at the expense of the stiffer (N2B) isoform (Nagueh et al., 2004). Similarly, an increased N2B-to-N2BA protein ratio has been reported in failing human DCM hearts (Makarenko et al., 2004). This resulted in reduced passive stiffness of isolated myofibrils. The authors further showed that, in DCM hearts, the contribution of titin to passive stiffness of cardiomyocytes was strongly reduced. This finding suggests that the dominance of the compliant titin isoform counteracts stiffness increases within the whole ventricle caused by increased fibrosis (Makarenko et al., 2004).

We still lack a detailed time course of stiffness changes under different cardiac pathological conditions. In contrast to the previous studies, research in a canine model revealed that the passive elastic modulus decreased by 41% shortly (1 h) after myocardial infarction. Unfortunately, this study lacked data from later timepoints (Forrester et al., 1972). These studies suggest that changes to myocardial stiffness need to be considered in the context of the progression state of a pathology. Hence, detailed studies are required to understand myocardial stiffness changes during the initiation and progression of specific cardiac diseases. Altogether, much will be learned from understanding the exact roles of intra- and extracellular molecular modifiers of tissue compliance and from characterizing different cardiac cell types modulating ECM stiffness to which collagen deposition strongly contributes. Functional studies are required to modulate different regions within diseased or injured cardiac tissue to better understand the extracellular mechanical changes that face the affected myocardium.

## Force Sensing and Transmission Within Cardiomyocytes

The heartbeat constantly exposes cardiomyocytes to physical forces by stress and strain. In the previous chapter, we discussed changes of ECM composition, titin phosphorylation, and cardiac tissue elasticity that occur within the diseased heart. These tissue stiffness changes are detected by cardiomyocytes and converted into gene expression changes. Here, we will focus on the machinery that is involved in these mechanosensitive signal transduction processes.

**FIGURE 1:**
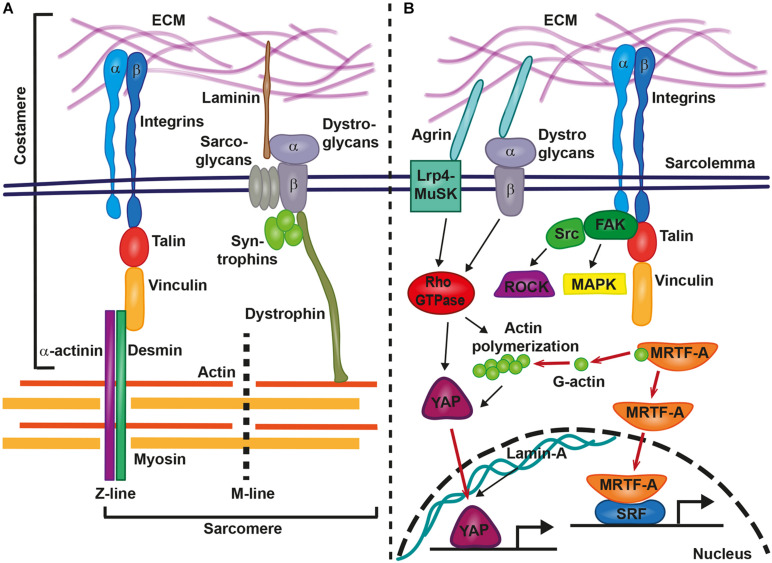
Involvement of costameres in mechanotransduction in cardiomyocytes. Costameres are composed of structural and signaling proteins. These structures provide the link between extracellular matrix (ECM) and the sarcomere **(A)** or result in the activation of various signaling cascades, that lead to the translocation of transcription factors to the nucleus and gene transcription changes **(B)**. Inside-out and outside-in signaling is mediated by integrins and the structural proteins talin, vinculin, α-actinin, and desmin provide the linkage from the sarcolemma to the sarcomere (actin, myosin, M-line, and Z-line). Similarly, The ECM-bound laminin connects via the dystrophin-glycoprotein complex with sarcomeric cytoskeletal components. Agrin acts via the Lrp4-MuSK or the dystrophin-glycoprotein complex on intracellular signaling cascades. Integrins recruit the focal adhesion kinase (FAK)-steroid receptor coactivator (Src)-complex, Rho-associated coiled-coil containing protein kinases (ROCKs) and mitogen-activated protein kinases (MAPKs). Rho-GTPase-induced actin polymerization liberates myocardin-related transcription factor A (MRTF-A) for nuclear translocation and supports the transport of YAP into the nucleus. Other relevant signaling cascades and proteins are described in the main text.

The mechanotransduction processes which translate mechanical stimuli into cellular signals are sensitive to changes in shear stress, cell adhesion forces, substrate rigidity, membrane or cytoskeletal stretching, and compression due to pressure. Focal adhesion integrin-based multi-protein complexes are crucial for these processes and mediate inside-out and outside-in signaling in response to mechanical stimuli or signals from the ECM or neighboring cells. Focal adhesions also couple the mechanical tension between ECM and the cytoskeleton (Iskratsch et al., 2014; Petridou et al., 2017). Within the heart, mechanical coupling of the extracellular space with the sarcomere is established by costameres, which are specialized focal adhesion protein complexes that connect the sarcolemma of cardiomyocytes to sarcomeric cytoskeletal components (Figure 1A) (Ervasti, 2003). Costameres are sites of adhesion and force transmission between cardiomyocytes and stabilize those sites that are impacted by lateral forces, thereby protecting the labile sarcolemma (Hersch et al., 2013). Costameres transmit cytoskeletal contractile forces that are passing across the sarcolemma, the ECM, and finally to neighboring cells. It has been hypothesized that this could be crucial for the uniform contraction of adjacent cardiomyocytes (Samarel et al., 2013). Similar to focal adhesion complexes in other cell types, costameres of the striated muscle contain structural proteins and vinculin is a main component together with talin, α-actinin, β1-integrin, and desmin, which is the physical link between Z-line and sarcolemma (Ervasti, 2003). Integrins are crucial for transmitting mechanical cues from the ECM to intracellular structures, via their adapter proteins talin, α-actinin, and vinculin (Figure 1A). These proteins act in a clutch-like manner and the outside-in transmission of mechanical forces depends on the composition and amount of these adaptor proteins (Elosegui-Artola et al., 2016). Interestingly, the presence of vinculin and β-integrins and their association with costameres is directly influenced by mechanical forces (Sharp et al., 1997). Costameres also include structural proteins of the dystrophin-glycoprotein complex, which includes dystrophin, sarcoglycans, dystroglycans, and syntrophins (Figure 1A). The dystrophin-glycoprotein complex physically links the ECM with the cytoskeleton (actomyosin networks). α-dystroglycan directly connects with the ECM protein laminin, whereas β-dystroglycan spans the membrane and is linked to the intracellular components of the dystrophin-glycoprotein complex (Peter et al., 2011) (Figure 1A).

Upon force transmission into cardiomyocytes, different biochemical downstream signaling pathways are involved in the induction of gene expression changes. At the costamere, integrins recruit and activate different signaling kinases, including focal adhesion kinase (FAK), Rho-associated coiled-coil containing protein kinases (ROCKs), and mitogen-activated protein kinases (MAPKs) (Samarel, 2005) (Figure 1B). *In vitro* studies on neonatal rat ventricular myocytes revealed the importance of FAK complex signaling for the response of cardiomyocytes to mechanical stretching. FAK became more strongly phosphorylated and changed its subcellular localization when cardiomyocytes were stretched (Torsoni et al., 2003). Signaling by FAK also regulated the activation of MEF2 and Jun-C in a model of mechanical stress within isolated rat ventricular myocytes (Nadruz et al., 2005). Mef2 and Jun-C had already been shown to activate a hypertrophic genetic program. As a response to mechanical stress, FAK signaling coordinated the activity of NF-kB in cardiomyocytes (Crosara-Alberto et al., 2009).

An involvement in mechanotransduction has also been described for the myocardin-related transcription factor A (MRTF-A/MKL1/MAL). Cellular and biochemical studies showed that the inactive form of MRTF-A is bound to cytoplasmatic globular actin (G-actin). It is released and translocated to the nucleus in a Rho-dependent manner when G-actin polymerizes to form actomyosin (Figure 1B) (Miralles et al., 2003; Kuwahara et al., 2010). There it acts as a co-transcription factor together with serum response factor (SRF) (Kuwahara et al., 2010). Hadden et al. (2017) showed that MRTF-A nuclear translocation depends on substrate stiffness and was highest at 20 kPa, which is near the physiological level of cardiac stiffness. Also, mechanical stretching of cardiomyocytes increased the nuclear presence of MRTF-A and mutations in MRTF-A attenuated the hypertrophic response to chronic pressure overload or angiotensin II infusion. This further revealed the importance of MRTF-A in mediating mechanical signaling in the hypertrophic heart (Kuwahara et al., 2010). A complete loss of both homologs, MRTF-A and MRTF-B, in mice caused a range of cardiac defects, including reduced contractility, sarcomere disarray and adult onset heart failure (Mokalled et al., 2015). In epicardial cells, MRTF-A translocation to the nucleus activated a cell motility program required for cell migration. The ablation of *Mrtfa* and *Mrtfb* impaired the development of the coronary microvasculature (Trembley et al., 2015).

The Hippo signaling pathway controls organ size and cell proliferation in response to mechanical tension (Meng et al., 2016). Two key players in this pathway are the transcriptional activator Yes-associated protein (YAP) and its interaction partner WW domain containing transcription regulator 1 (WWTR1; also known as TAZ). These proteins are involved in sensing and transmitting mechanical signals to the nucleus to regulate specific gene expression. Mechanotransduction via YAP/TAZ occurs through Rho GTPase activity and tension of the actomyosin cytoskeleton (Dupont et al., 2011) (Figure 1B). Dupont et al. (2011) showed that YAP/TAZ was nuclear on stiff substrates whereas it became cytoplasmic on softer substrates. This activation of the YAP pathway was dose-dependent. The levels of YAP nuclear localization increased following a sigmoidal curve in human adipose-derived stem cells that had been plated on hydrogels with stiffness gradients ranging from 2 to 40 kPa (Hadden et al., 2017). This finding was conflicting with the previous study that had reported a switch-like manner of YAP nuclear localization depending on specific stiffness values (Dupont et al., 2011). YAP localization to the nucleus increased in a linear manner when stiffness was in the range between 12 and 20 kPa, whereas a plateau of YAP nuclear localization was reached when substrate stiffness was 20–40 kPa (Hadden et al., 2017). Hence, further studies are required to elucidate the precise regulatory mechanism of YAP localization in response to tissue stiffness.

While YAP is an important regulator and inducer of cardiomyocyte proliferation in the embryonic and postnatal heart, it does not induce hypertrophic growth of the myocardium. Strikingly, nuclear YAP/TAZ localizes to the nucleus of the infarcted murine myocardium at 3, 5, 14, and 36 days post myocardial infarction but not in remote, non-injured regions (Mosqueira et al., 2014). In human cardiomyocyte progenitor cells, localization of YAP depended on the cytoskeleton and on myosin contractility. YAP nuclear localization strongly increased in human cardiomyocyte progenitor cells that were cultured on collagen- and fibronectin-coated polyacrylamide gels with a Young’s modulus higher than 10 kPa when compared to low stiffness gels (0.5–0.7 kPA). When YAP/TAZ was silenced, human cardiomyocyte progenitor cells adhered less to stiff substrates (>10 kPa), expression of genes involved in cell matrix interactions changed, and their migration capacity was reduced. Yet, no differences of these parameters were observed when YAP/TAZ-silenced human cardiomyocyte progenitor cells were cultured on soft gels. Human cardiomyocyte progenitor cells cultured on fibronectin-coated polyacrylamide gels with standard heart stiffness (10kPa) expressed the cardiac differentiation program (GATA-4, NKX2.5). However, this was prevented by YAP/TAZ silencing and cells instead expressed genes that indicated a commitment to the endothelial lineage (Mosqueira et al., 2014). Similarly, in fibroblasts, YAP-signaling was activated upon myocardial infarction and enabled differentiation and ECM gene expression via MRTF-A (Francisco et al., 2020). This pointed at the potent role of YAP/TAZ in progenitor cells to induce specific cellular fates in response to extracellular mechanical stimuli. This may have major implications on the healing capacity of injured tissues when mechanical properties have changed.

The mode by which YAP/TAZ becomes activated by mechanical stiffness has raised much interest. This led to the discovery of the ECM protein agrin as a stiffness sensor involved in YAP pathway activation. Its function is directly dependent on matrix stiffness and mediates YAP signaling in cells surrounded by hard but not soft matrices. While this has only been shown in mouse hepatocarcinoma cells (Chakraborty et al., 2017), it is conceivable that agrin may act in a similar manner in cardiomyocytes. In tune with such a model, agrin is required for cardiomyocyte proliferation and cardiac regeneration in the neonatal mouse heart in a way that involves YAP- and ERK signaling. A very exciting finding related to agrin is its involvement in the regeneration of adult murine cardiomyocytes. In a model of myocardial infarction, adult mice treated with recombinant agrin showed cardiomyocyte proliferation, a reduction in scarring, and improved cardiac function at 35 days post infarction. When treated with agrin, sarcomere disassembly was induced in P7 cardiomyocytes in culture and the expression of the sarcomeric protein cardiac troponin T was prevented. Interestingly, agrin application led to increased nuclearization of YAP in cardiomyocytes 1 day after myocardial infarction and the inhibition of YAP prevented agrin-induced cardiomyocyte proliferation (Bassat et al., 2017). This suggested a role of agrin as a modulator of cardiomyocyte differentiation, proliferation, and regeneration in a way that involves YAP signaling. The implications of this exciting discovery have been discussed in more detail in a recent review (Bigotti et al., 2020). Altogether the YAP/TAZ pathway plays a crucial role in cardiac mechanotransduction and is a potent regulator of cell fate and function.

The nuclear envelope has received much attention for its role in mediating mechanical stress into the nucleus. The structural nuclear envelope protein lamin A is stiffness-dependent and regulates chromatin organization, gene expression, and DNA replication (Carmosino et al., 2014). It has been suggested that lamins determine lineage specification toward contractile and hard tissues (Carmosino et al., 2014). Indeed, protein levels of lamin A are higher when organ stiffness increases in mice and men (Swift et al., 2013). Increasing substrate stiffness caused an exponential increase of lamina A in human adipose-derived stem cells (Hadden et al., 2017). One way by which lamin A affected mechanosensitive signaling was by promoting the translocation of YAP to the nucleus and by positively regulating the SRC pathway, which controls genes related to sarcomeric assembly and function (Balza and Misra, 2006; Carmosino et al., 2014). Patients and mice with mutations in lamin A suffer from cardiac diseases, including DCM. In murine knockout models, cardiomyocytes were less resistant to mechanical tension with reduced nuclear stability and increased rates of apoptosis (Nikolova et al., 2004).

These findings highlight the complex regulation involved in cardiac mechanotransduction. The sensation of physical changes and mechanotransduction within cardiomyocytes involves the costamere, a specialized complex of focal adhesion proteins, which mediates inside-out and outside-in signaling. Also, several downstream mechanosensitive signaling pathways become activated in cardiomyocytes in response to changes of matrix stiffness. These include the nuclear sensors YAP/TAZ, lamin A, and MRTF. These studies showed that general mechanisms of mechanotransduction signaling also function in cardiomyocytes and are required for adequate responses to physical changes and during differentiation.

## Consequences of Stiffness Changes on Cardiomyocyte Performance

**FIGURE 2:**
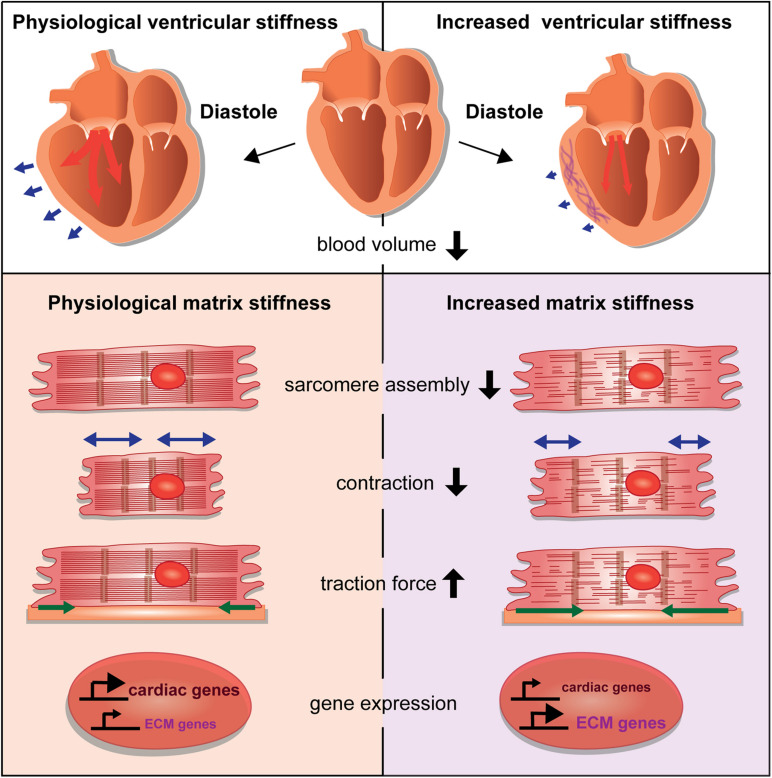
Consequences of stiffness changes for cardiomyocytes. The increase of ventricular cardiac stiffness results in reduced diastolic blood volume. Blue arrows indicate the ventricular diastolic expansion. A stiffer matrix can interfere with sarcomere assembly and leads to changes in cardiomyocyte behavior by affecting cellular contraction and traction forces. Changes in matrix stiffness also cause aberrant gene expression that alters the balance of cardiac versus ECM genes.

Changes of the cardiac ECM affect the compliance of the cardiac ventricle, which impacts the diastolic volume (Figure 2). What are the consequences on cardiomyocytes when such tissue stiffness changes occur due to cardiac injury or disease? It has been a hallmark discovery that changes in substrate stiffness can trigger dramatic changes in the morphology, behavior, and differentiation state of cells. Mesenchymal stem cells were differentiated into a range of divergent neurogenic, myogenic, or osteogenic tissues simply by modulating their substrate stiffness on which they had been plated (Engler et al., 2006). These experiments revealed the importance of biomechanical signaling for cell fate determination and raised increasing interest in the responses of cardiomyocytes to extracellular stiffness changes. Cardiomyocytes cultured on substrates with elasticity values representing physiological and diseased cardiac stiffness or with gradients of stiffness developed differently (Hadden et al., 2017; Chin et al., 2019). For instance, cardiomyocytes cultured on a stiff, fibrotic tissue-like matrix lacked striated myofibrils and failed to beat properly (Engler et al., 2008).

Heras-Bautista et al. used polyacrylamide (PAA) hydrogel matrices with ranges of stiffness corresponding with embryonic (12 kPA), adult (30 kPA), or fibrotic (123 kPA) cardiac tissue as substrates for murine induced pluripotent stem cell-derived cardiomyocytes. Cardiomyocytes that were grown on a stiff matrix had an impaired contractile function with disarranged sarcomeres whereas cardiomyocytes grown on soft and medium matrices had highly organized sarcomeres. Soft matrix conditions also resulted in comparative transcriptomes in contrast to cardiomyocytes exposed to a stiff hydrogel. Gene expression analyses of cardiomyocytes grown on stiff matrices revealed many deregulated genes, especially of genes related to developmental programs (Heras-Bautista et al., 2019). In addition, cardiomyocytes grown under stiff matrix conditions also upregulated different ECM-components or modulators, such as the collagens Col1a1, Col1a2, Col4a2, Col6a3, and Col8a2, matrix metalloproteinases, inhibitors of matrix metalloproteinases, and tenascinC, (Heras-Bautista et al., 2019). Similarly, bovine and murine adult cardiac side population progenitor cells were cultured on substrates with a stiffness corresponding with that of physiological or fibrotic myocardium. This study revealed that increased tissue stiffness not only augmented proliferation and cell cycling, reduced myocardial differentiation based on the levels of α-actinin expression, and accelerated cell aging as indicated by a reduction in telomere length, but also caused an increased expression of genes encoding for ECM and adhesion proteins (Qiu et al., 2015). Hence, stiff matrix conditions induced cardiomyocytes and progenitors to further change the matrix composition.

Culturing embryonic rat cardiomyocytes on substrates representing physiological or fibrotic stiffness values revealed that most cells were angular in shape on stiff substrates and roundish on soft substrates. However, neither myofibril organization and function, nor costamere numbers were affected by substrate stiffness (Hersch et al., 2013). Traction force microscopy of cells cultured on substrates with different stiffness values revealed an increased force generation by cardiomyocytes that corresponded with increasing substrate stiffness. This suggested a potent mechanism of force generation by cardiomyocytes, which ensures the stable cell contraction independently of substrate stiffness (Hersch et al., 2013).

Ribeiro et al. examined human pluripotent stem cell-derived cardiomyocyte behaviors when cultured on substrates with a stiffness ranging from 4 to 100 kPA. Contractile forces increased on very stiff matrices (90 kPA), which was in agreement with a previously mentioned study (Hersch et al., 2013). In contrast, cell shortening was reduced in cardiomyocytes compared to that of cells grown on substrates with a physiological stiffness (21 kPa). Hence, the authors suggested that very stiff matrices restrain cell shortening (Ribeiro et al., 2020).

Those studies revealed the importance of a cardiac matrix with physiological stiffness values. Aberrations can dramatically change cardiomyocyte behavior and performance by influencing differentiation, contractile function, gene expression, cell morphology, and force generation (Figure 2). How mechanotransductive pathways induce such molecular changes has raised increasing attention. Santos et al. utilized rat ring-shaped engineered connective tissue comprised of cardiac fibroblasts and collagen I while using ROCK signaling inhibitors. This resulted in reduced tissue stiffness, and also reduced TGF-β signaling-driven tissue stiffening (Santos et al., 2019). In this context, the inhibitory effect of ROCK inhibitors on stiffness was mediated by the regulation of lysyl oxidase, the collagen cross-linking enzyme and involved the activation of the actin-dependent MRTF/SRF pathway. This study also showed, that ROCK inhibitors similarly decreased stiffness of human engineered connective tissue and rat engineered cardiac muscle (Santos et al., 2019).

In a related study, Pandey et al. sought to address how cardiomyocytes probe and sense their environment. For this, they cultured neonatal rat cardiomyocytes on nanopillar arrays with different levels of stiffness. This revealed that the induction of cardiomyocyte hypertrophy on a stiff matrix was dependent on protein kinase C (PKC). PKC regulated the non-receptor tyrosine kinase Src, which activated non-muscle myosin contractions at cell edges that are involved in sensing of cell rigidity. By using a tension sensor for the costamere adaptor protein talin, the authors revealed that cyclic stretching of talin is induced downstream of PKC and Src under conditions of physiological stiffness but is continuously induced on matrices representing fibrotic stiffness. In infarcted mouse hearts, a model for DCM, PKCδ and PKCα localized to integrin adhesion sites at costameres together with non-muscle myosin light chain. This suggested an involvement of PKC- and Src-induced non-muscle myosin contractility for rigidity sensing in the diseased myocardium (Pandey et al., 2018).

Another recent study provided further insights into the complex relationship, upon cardiac infarction, between ventricular stiffness, ECM protein availability, YAP signaling activation, and induction of cardiomyocyte proliferation (Wang et al., 2020). The authors applied fetal ECM proteins and modified the stiffness of neonatal mouse hearts after myocardial infarction at day 5. Cardiac function improved three weeks post-surgery in comparison to non-treated hearts when they applied fetal ECM and decreased tissue stiffness by inhibiting lysyl oxidase-induced collagen crosslinking (inhibition by BAPN). Reduced stiffness due to lysyl oxidase inhibition also significantly diminished scarring. In a comparable experiment, mouse cardiac explants were cultured from day 1 under conditions of BAPN- or ribose-treatment to reduce or increase stiffness, respectively (Wang et al., 2020). These experiments revealed that a reduction of stiffness together with fetal ECM treatment increased cardiomyocyte proliferation, reduced collagen deposition, and augmented vascularization. A softer matrix increased the number of cardiomyocytes that exhibited nuclear YAP. This observation apparently contrasts with the study by Mosqueira et al., which reported increased levels of nuclear YAP in human cardiomyocyte progenitor cells grown on stiff matrices. These differences may result from using cardiomyocyte progenitor cells versus explants of infarcted cardiac tissue. Hence, additional studies are required to clarify the effects of stiffness changes on YAP nuclear localization. However, both studies reported an increase of cardiomyocyte proliferation upon nuclear YAP localization (Mosqueira et al., 2014; Wang et al., 2020). Softer matrix conditions in addition to a fetal ECM treatment led to an increased expression of agrin (Wang et al., 2020). Agrin promotes cardiomyocyte proliferation as described above and this may contribute to regenerative effects as well (Bassat et al., 2017). Hence, a softer matrix in combination with embryonic soluble ECM factors had positive effects on cardiomyocyte proliferation, which involved YAP-signaling and actin stability (Wang et al., 2020). These findings impressively illustrated the importance of mechanical cues in regulating regenerative processes of the heart.

Much of our knowledge related to the cellular changes in cardiomyocytes in response to increased tissue stiffness has come from *in vitro* studies. These enabled researchers to use well-defined experimental modulations. Several recent studies revealed direct cellular effects due to matrix stiffness changes. Yet, we are far from understanding the detailed interplay between cardiac stiffness and mechanotransduction in the context of the diseased or regenerating heart.

## Discussion

Cardiac diseases often result in fibrotic tissue deposition or cardiomyocyte hypertrophy, two irreversible physiological changes that have a major impact on cardiac function. In this review, we focused on changes of cardiac stiffness that occur in diseased hearts. Myocardial stiffness, which is the potential of cardiomyocytes to resist contraction-induced elongation or shortening, relies on intra- and extracellular components. Stiffness increases in diseased hearts often result from alterations of ECM components. For instance, ECM becomes deposited during fibrosis after myocardial infarction (Frangogiannis, 2019). Fibroblasts are the main players that cause an enhanced ECM deposition. Currently, the roles of other cardiac cell types in depositing and modeling the fibrotic ECM and thus influencing cardiac stiffness have not systematically been addressed. A recent study revealed that macrophages contribute to collagen deposition in the injured mouse and zebrafish heart (Simões et al., 2020). Yet, how macrophages impact myocardial tissue stiffness upon cardiac insult still needs to be resolved.

When the diseased heart turns less compliant, stiffness of the ventricular myocardium prevents that the required physiological volume during diastole is reached and blood flow throughout the body is insufficient. However, besides the global impact that an increase of myocardial stiffness has on heart physiology, there are also crucial cellular changes that occur within the affected myocardium. Here, we reviewed the sophisticated molecular machinery that senses stiffness changes in the environment of cardiomyocytes and mediates appropriate responses (Figure 1). Mechanotransductive signaling cascades activate gene expression changes with wide consequences for cardiomyocyte function, differentiation, and contractility (Figure 2). This may directly affect intracellular components such as titin that are required for cardiomyocyte passive stiffness. We still need a better understanding why cardiomyocytes show such prominent responses to biomechanical changes. What are short- and long-term consequences of such functional modifications? Answers to these questions may have relevance for a better understanding of cardiac diseases. We need to know, what occurs to cardiomyocytes suddenly facing a different, stiffer environment. Here, we reported that this can cause changes in sarcomere assembly and contractility, aberrant ECM production, cell shape changes, and modifications in force generation. Moreover, stiffness changes also affect the differentiation state of cardiomyocytes. This means, that beside the immense loss of cardiomyocytes after myocardial infarction, remaining cardiomyocytes may lose functionality with further debilitating consequences on heart performance. Although, recent advances showed that cardiomyocytes can sense and respond to those changes, we actually lack detailed studies of such mechanisms. What are the consequences of fibroblast-driven fibrotic tissue deposition and could this even worsen cardiac function after a myocardial infarction? While the deposition of a rigid fibrotic scar is fatal for long-term ventricular functionality, it is indispensable to prevent the heart from ventricular rupture and for maintaining the structure of the heart shortly after a myocardial infarction (Frangogiannis, 2019).

Knowledge about how cardiomyocytes respond to pathological tissue stiffness changes is crucial when it comes to the development of regenerative therapies. External factors that modulate the ECM and tissue stiffness in the diseased heart represent an excellent source for drug and treatment development. They are more easily applicable as part of a therapy than drugs that directly target an intracellular signaling cascade. *In vitro* studies have potently shown that changing substrate stiffness affects cardiomyocyte function. However, there are only few reports about how stiffness changes may lead to cardiomyocyte de-differentiation and increased proliferation, which is required for cardiac repair. We are just beginning to discover powerful molecules for inducing cardiomyocyte proliferation and to test their potential *in vivo* in the diseased heart. One pioneering study involved the discovery of the ECM protein agrin, a stiffness sensor in the mechanotransduction cascade regulating cardiomyocyte proliferation (Bassat et al., 2017; Chakraborty et al., 2017). Its potential to improve fibrosis, cardiac function and adverse remodeling after myocardial infarction was recently even shown in big animal models (Baehr et al., 2020).

Another promising approach based on modulating isoforms of the basement membrane protein laminin interfered with myocardial stiffness in HFpEF patients (Hochman-Mendez et al., 2020). Laminin is part of the costamere (Figure 1A) and involved in transmitting extracellular biomechanical signals to the contractile apparatus. Altering the ECM laminin content affected titin isoform expression, which regulates passive stiffness of cardiomyocytes (Hochman-Mendez et al., 2020). Such novel approaches promise the development of cardiac therapies after myocardial infarction.

However, besides this amplitude of recent discoveries about the mechanotransduction machinery in cardiomyocytes and the consequences of aberrant tissue stiffness, many questions still remain unanswered. Little is known about the prospects of therapeutic modifications of cardiac ECM and its stiffness, in order to improve cardiomyocyte performance and induce regenerative processes without softening of the ventricular wall. And how exactly can costamere proteins detect substrate stiffness? How does aberrant mechanical signaling due to a stiff matrix interfere with cell migration, intercellular communication, and ligand availability for cardiomyocytes and other cardiac cell types? Several studies have shown the importance of the ECM for cytokine- and growth factor-mediated signaling (Frangogiannis, 2017). It also needs to be addressed, whether other cell types respond to tissue stiffness changes in the heart and how this affects myocardial regeneration. For example macrophages are highly responsive to matrix stiffness changes (Sridharan et al., 2019) and also cause stiffness increases and diastolic dysfunction (Hulsmans et al., 2018). Many of those questions have only been addressed to a limited extent. However, their answers are crucial when it comes to the development of novel regenerative therapies that target tissue stiffness changes in the heart.

## Author Contributions

JM established a plan for this manuscript and designed the figures. JM and SA-S wrote the draft and approved the submitted manuscript. Both authors contributed to the article and approved the submitted version.

## Conflict of Interest

The authors declare that the research was conducted in the absence of any commercial or financial relationships that could be construed as a potential conflict of interest.
